# Effect of Ceramides Derivatives from the Peach on Skin Function Improvement in UV-Irradiated Hairless Mice

**DOI:** 10.3390/foods13233824

**Published:** 2024-11-27

**Authors:** Jinhee Kim, Minhee Lee, Wonhee Cho, Eunhee Yoo, Jinhak Kim, Yuri Gwon, Musashi Okayasu, Jeongmin Lee

**Affiliations:** 1 Department of Medical Nutrition, Kyung Hee University, Yongin 17104, Republic of Korea; jinhee625@khu.ac.kr (J.K.); miniclsrn@khu.ac.kr (M.L.); wonhi1117@khu.ac.kr (W.C.); a01089219455@gmail.com (E.Y.); 2 Department of Food Innovation and Health, Kyung Hee University, Yongin 17104, Republic of Korea; 3 R&D Division, Daehan Chemtech Co., Ltd., Gwacheon-si 13840, Republic of Korea; jhkim@dhchemtech.com (J.K.); rnd@dhchemtech.com (Y.G.); 4 OKAYASU Co., Ltd., 1004-2 Hirakata, Koshigaya City 343-0002, Japan; musashi@okayasu1935.co.jp

**Keywords:** ceramide, peach, UV, skin health, hairless mice

## Abstract

This study investigated the protective effects of a ceramides derivates from the peach (PF3) on photoaging by UV-irradiated hairless mice. Mice were randomly divided into seven groups: AIN93G without UVB exposure (normal control, NC), AIN93G with UVB exposure (control, C), AIN93G supplemented 100 mg/kg body weight (BW) of L-ascorbic acid with UVB exposure (AA), AIN93G supplemented 100 mg/kg BW of arbutin with UVB exposure (Arbutin), AIN93G supplemented 10 mg/kg BW of PF3 with UVB exposure (10PF3), AIN93G supplemented 20 mg/kg BW of PF3 with UVB exposure (20PF3), and AIN93G supplemented 40 mg/kg BW of PF3 with UVB exposure (40PF3). The study examined the impact of PF3 on skin hydration, wrinkle formation, and melanogenesis using enzyme-linked immunosorbent assay (ELISA), real-time polymerase chain reaction (real-time PCR), and Western blot analysis. The PF3 demonstrated significant protective effects against photoaging by reducing skin wrinkle formation, decreasing epidermal and dermal thickening, and improving skin hydration. It also enhanced the expression of moisture-related factors (hyaluronic acid synthase [HAS], long-chain ceramides [LCBs], dihydroceramide desaturase 1 [DEGS1], and type I collagen [COL1A]) and antioxidant enzyme activities while reducing pro-inflammatory cytokines and oxidative stress markers. The PF3 supplementation positively modulated skin wrinkle formation-related factors, increasing collagen-related gene expression and decreasing matrix metalloproteinases. Additionally, PF3 showed potential in regulating melanogenesis by reducing the nitric oxide and cAMP content, as well as the expression of melanogenesis-related proteins. These comprehensive findings suggest that PF3 supplementation may be an effective strategy for preventing and treating UVB-induced skin photoaging through multiple mechanisms, including improved skin structure, hydration, antioxidant defense, and reduced inflammation and pigmentation.

## 1. Introduction

The skin, as the body’s largest organ, plays a crucial role in protecting the body from external threats and maintaining overall health. In recent years, there has been a growing interest in skin health, not only from a medical perspective but also in the realms of cosmetics and wellness. This increased attention stems from a deeper understanding of the skin’s complex functions and its impact on general well-being [1,2]. Ultraviolet (UV) radiation from the sun is a well-known environmental factor that can significantly impact skin health and appearance [3,4]. Prolonged exposure to UV radiation could lead to various detrimental effects, including decreased skin hydration, the formation of wrinkles, and uneven skin pigmentation. UV radiation could disrupt the skin’s natural barrier function, leading to increased transepidermal water loss (TEWL) and overall dull complexion [5,6,7]. Moreover, UV-induced oxidative stress can damage cellular components, further contributing to the breakdown of the skin’s extracellular matrix and the development of wrinkles. Uneven skin pigmentation, often manifested as age spots or sun-induced freckles, is another common consequence of UV exposure. UV radiation can stimulate the production of melanin, the pigment responsible for skin color, leading to the appearance of darker patches on the skin. Additionally, chronic UV exposure has been linked to more severe skin conditions, including various forms of skin cancer, emphasizing the importance of understanding and mitigating its effects [8,9,10].

Ceramides are essential components of the intercellular lipids of the stratum corneum. Composed of a sphingoid base and a fatty acid, ceramides are crucial for the formation of the skin’s protective barrier. These lipid molecules play a vital role in maintaining the skin’s barrier function and preventing TEWL. While ceramides can be synthesized endogenously within the human body, they can also be obtained from various plant sources, such as rice, wheat, and vegetables. These plant-derived ceramides have gained increasing attention in the cosmetic and dermatological industries due to their potential benefits for skin health and appearance [11,12,13,14].

Previous studies of ceramide supplements in food have shown that ceramides’ (glycosylceramides) improved permeability barrier function and water retention [15,16,17]. Among various plant sources, peaches (Prunus persica) stand out for their high ceramide content, potentially offering a more reliable and consistent source compared to other plants. This abundance of ceramides in peaches makes them an attractive subject for research in skin protection and health. Recent research has explored the ceramide profiles of different peach cultivars, revealing significant variations in both concentration and composition. These differences among peach varieties present intriguing possibilities for skincare applications. Some cultivars have been found to contain higher proportions of long-chain ceramides, which are particularly effective in reinforcing the skin’s barrier function. Others exhibit a more diverse range of ceramide subclasses, potentially offering a broader spectrum of skin benefits. Factors such as growing conditions, ripeness at harvest, and post-harvest processing can influence the ceramide content and quality in peaches. This variability among peach cultivars offers both challenges and opportunities for researchers and formulators in the skincare industry. By carefully selecting and blending ceramides from different peach varieties, it may be possible to create more targeted and effective skincare products. Furthermore, this knowledge could lead to the development of specialized peach cultivars bred specifically for their superior ceramide profiles, potentially revolutionizing natural skincare ingredients [18,19].

In this study, we investigated whether ceramide derivatives from peaches could protect the skin against UV exposure in SHK-I hairless mice. Our aim was to understand the mechanisms underlying this potential protective effect and explore the possibilities of using peach-derived ceramides in skincare formulations. This research not only contributes to our understanding of skin biology and UV protection but also has potential implications for the development of novel, natural approaches to skincare and UV damage prevention.

## 2. Materials and Methods

### 2.1. Ceramides Preparation

Ceramide derivatives from the peach ceramide (PF3) were provided by Daehan Chemtech Co., Ltd. (Gwacheon, the Republic of Korea). The raw material of peach ceramide was prepared by removing the peel and seeds and then squeezing. The crude peach oil was extracted using n-hexane with raw material, and then, the solvent was removed. After that, it was extracted with ethanol, concentrated to remove the ethanol, cyclodextrin was added, and dried to obtain peach ceramide (PF3). The powders were kept at −20 °C until the experiments.

### 2.2. Experimental Design

Fifty-six SKH-1 hairless mice (male, 5 weeks old) were purchased from SaeRon Bio (Uiwang, the Republic of Korea). The mice were accommodated in an automatically managed conditions room (40~60% relative humidity, 12/12 h light/dark cycle, and at 22 ± 2 °C). The mice were split into seven groups of eight animals each as follows: AIN93G without UVB exposure (normal control, NC), AIN93G with UVB exposure (control, C), AIN93G supplemented 100 mg/kg body weight (BW) of L-ascorbic acid with UVB exposure (AA; Sigma-Aldrich, St. Louis, MO, USA), AIN93G supplemented 100 mg/kg BW of arbutin with UVB exposure (Arbutin; Sigma-Aldrich), AIN93G supplemented 10 mg/kg BW of PF3 with UVB exposure (10PF3), AIN93G supplemented 20 mg/kg BW of PF3 with UVB exposure (20PF3), and AIN93G supplemented 40 mg/kg BW of PF3 with UVB exposure (40PF3). All groups, except NC, were exposed to UVB three times per week using a UVB lamp (Sankyo Denki Co., Yokohama, Japan). The UVB intensity started at 150 mJ/cm^2^ (1 week) and progressively increased to 300 mJ/cm^2^ (2 week), 450 mJ/cm^2^ (3 week), and 600 mJ/cm^2^ (4 to 8 week). The experiments were permitted by the Institutional Animal Care and Use Committee of Kyung Hee University (KHGASP-23-089).

### 2.3. Measurement of Skin Hydration and Histological Observation

The hydration, thickness, and wrinkle formation of the dorsal skin were measured using the methods in the previous study [20,21]. The hydration of the dorsal skin surface was measured using Howskin (Seoul, the Republic of Korea), while the thickness and wrinkle formation of the dorsal skin were measured by histological observation. Dorsal skin tissues were stained with hematoxylin and eosin and examined under a light microscope.

### 2.4. Measurement of Antioxidant Enzyme Activities, Tyrosinase, Nitric Oxide (NO), and Cyclic Adenosine Monophosphate (cAMP)

The antioxidant enzyme activities, tyrosinase, NO, and cAMP activities of dorsal skin tissue, were measured using the following kits: SOD (Superoxide dismutase 1 ELISA kit, Biovision Inc., Milpitas, CA, USA), CAT (Catalase Activity Colorimetric/Fluorometric Assay kit, Biovision Inc.), GPx (Glutathione peroxidase Activity Colorimetric Assay Kit, Biovision Inc.), tyrosinase (Tyrosinase activity Assay Kit, Abcam, Cambridge, UK), the levels of NO (Nitric Oxide Assay Kit, Abcam), and cAMP (cAMP ELISA kit, Enzo Life Sciences, Plymouth Meeting, PA, USA). All measurements were performed according to the manufacturers’ manuals.

### 2.5. Isolation of Total RNA and Real-Time Polymerase Chain Reaction (PCR)

Total RNA was isolated from dorsal skin tissue using a RNeasy extract kit (Qiagen, Gaithersburg, MD, USA), then RNQ quantification, cDNA synthesis, and real-time PCR analysis were performed to assess the expression of the genes HAS1-3, LCB1 (SPT), DEGS1, Fibrillin-1, and GAPDH, following the methods described in a previous study [20,21].

### 2.6. Isolation of Protein and Western Blot Analysis

Dorsal skin tissues were lysed using the CelLytic^TM^ MT Cell Lysis Reagent (Sigma-Aldrich) to which the Halt^TM^ Protease & Phosphatase inhibitor Cocktail (Thermo Fisher Scientific, Rockford, IL, USA) was added. Homogenized samples were centrifuged at 14,000 rpm at 4 °C for 20 min. the protein content was quantified using the Bradford assay. Protein samples (50 μg each) were loaded onto 10% Mini-PROTEAN^®^ TGX^TM^ Precast Gels (Bio-Rad, Hercules, CA, USA) and transferred to membranes using the Trans-Blot^®^ Turbo^TM^ Transfer system (Bio-Rad). Membranes were blocked with buffer (5% skim milk in Tris-buffered saline with 1% Tween^®^ 20) for 1 h at room temperature. After washing, the membranes were exposed overnight at 4 °C to the following primary antibodies: CerS4 (LASS4), IκBα, phosphor-IκBα, p65, phosphor-p65, COX-2, Smad3, phosphor-Smad3, Collagen type 1, MMP-1, MMP-2, MMP-3, MMP-9, JNK, phosphor-JNK, c-Fos, phosphor-c-Fos, c-Jun, phosphor-c-Jun, PKA, CREB, phosphor-CREB, MITF, TRP-1, TRP-2, and β-actin. Washed membranes were incubated with secondary antibodies (anti-rabbits/mouse/Goat IgG HRP conjugated, 1:5000) for 1 h at room temperature. Antibodies were purchased from Abcam (Cambridge, MA, USA), Bethyl (Montgomery, TX, USA), Cell Signaling Technology (Beverly, MA, USA), or LSbio (Seattle, WA, USA). Washed membranes were exposed to the luminol substrate EzWestLumi plus (ATTO, Tokyo, Japan), and luminescence was captured using Ez-Capture II equipment (ATTO). Images were analyzed using CS Analyzer 3.0 software (ATTO).

### 2.7. Statistical Analysis

All results were shown as the mean ± standard deviation (SD). The data were statistically determined using Duncan’s multiple range tests after one-way analysis of variance (ANOVA) using SPSS (IBM SPSS Statistic v.25.0, IBM Corp., Armonk, NY, USA). Differences were considered statistically significant at *p* < 0.05.

## 3. Results

### 3.1. Effects of PF3 on Skin Wrinkle Formation and Epidermal and Dermal Thickness in UVB Exposed SKH-I Hairless Mice

**Figure 1:**
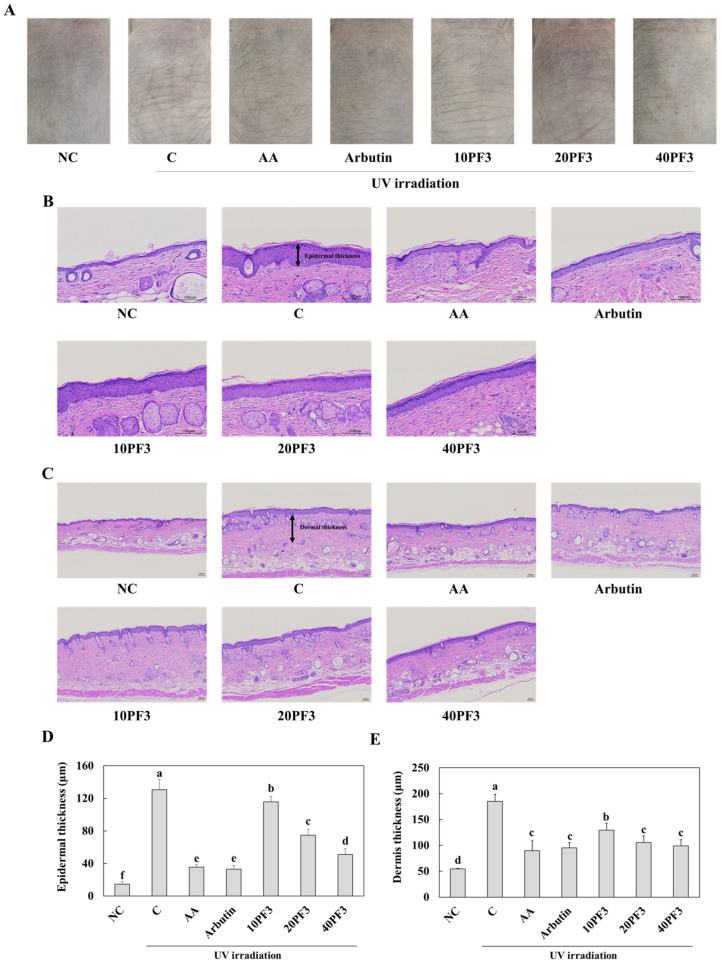
Effect of PF3 on morphological (**A**) and histopathological changes (**B**,**C**) (epidermal (**D**) and dermal thickness (**E**)) in the dorsal skin of UVB-irradiated SKH-I hairless mice. NC, AIN93G without UVB irradiation; C, AIN93G with UVB irradiation; AA, AIN93G supplemented 100 mg/kg body weight (BW) of L-ascorbic acid with UVB irradiation; Arbutin, AIN93G supplemented 100 mg/kg BW of arbutin with UVB irradiation; 10PF3, AIN93G supplemented 10 mg/kg BW of PF3 with UVB irradiation; 20PF3, AIN93G supplemented 20 mg/kg BW of PF3 with UVB irradiation; 40PF3, AIN93G supplemented 40 mg/kg BW of PF3 with UVB irradiation. Data represent the mean ± SD (*n* = 8). Different letters (a–f) represent significant differences at *p* < 0.05, as determined by Duncan’s multiple rang test.

To investigate the effect of PF3 on skin wrinkle formation, the epidermal and dermal thickness of the dorsal skin of UVB-irradiated SHK-I hairless mice were studied. Skin wrinkles were increased in the control (C) group compared to the normal control (NC) group. The L-ascorbic acid (AA), arbutin, and PF3 supplement groups showed reduced skin wrinkles induced by UVB irradiation (Figure 1A). The epidermal and dermal thickness were significantly increased in the C group compared to the NC group; however, the oral administration of AA, arbutin, and PF3 groups had significantly decreased epidermal and dermal thickness compared to the C group (*p* < 0.05) (Figure 1B,C). These finding suggest that the PF3 supplement improved skin wrinkle formation and epidermal and dermal thickness induced by UVB irradiation.

### 3.2. Effects of PF3 on Skin Hydration and mRNA Expression of Skin Moisturizing-Related Factors in UVB-Irradiated SKH-I Hairless Mice

**Figure 2:**
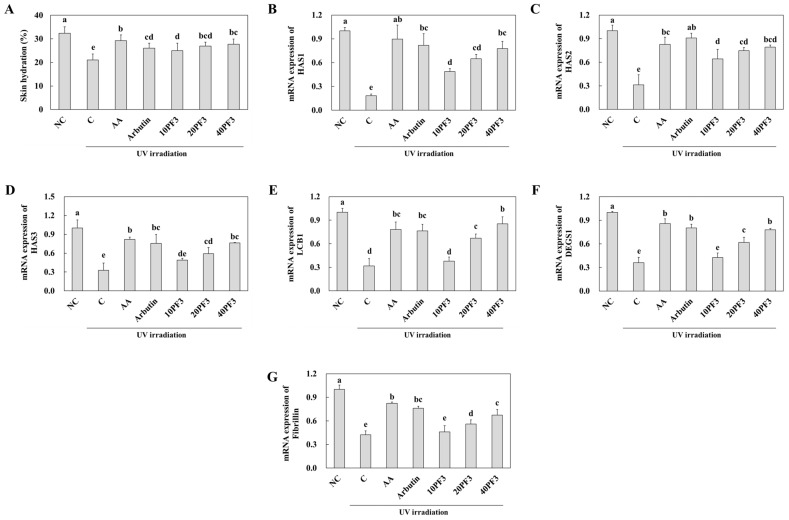
Effect of PF3 on skin hydration (**A**) and mRNA expression of HAS1 (**B**), HAS2 (**C**), HAS3 (**D**), LCB1 (**E**), DEGS1 (**F**), and Fibrillin-1 (**G**) in the dorsal skin of UVB-irradiated SKH-I hairless mice. NC, AIN93G without UVB irradiation; C, AIN93G with UVB irradiation; AA, AIN93G supplemented 100 mg/kg body weight (BW) of L-ascorbic acid with UVB irradiation; Arbutin, AIN93G supplemented 100 mg/kg BW of arbutin with UVB irradiation; 10PF3, AIN93G supplemented 10 mg/kg BW of PF3 with UVB irradiation; 20PF3, AIN93G supplemented 20 mg/kg BW of PF3 with UVB irradiation; 40PF3, AIN93G supplemented 40 mg/kg BW of PF3 with UVB irradiation. Data represent the mean ± SD (*n* = 8). Different letters (a–e) represent significant differences at *p* < 0.05, as determined by Duncan’s multiple rang test.

We investigated the effect of PF3 on the skin hydration and mRNA expression of skin moisture-related factors of the dorsal skin of UVB-irradiated SHK-I hairless mice. Skin hydration was significantly decreased in C group compared to NC group. AA, arbutin, and PF3 supplement groups were significantly increased in skin hydration (*p* < 0.05) (Figure 2A). Moreover, mRNA expression of HAS1-3 was significantly decreased in C group compared to NC group. AA, arbutin, and PF3 supplement groups increased in that compared to C group (*p* < 0.05) (Figure 2B–D). mRNA expression of LCB1, DEGS1, and Fibrillin-1 was significantly decreased in C group than NC group; however, AA, arbutin, and PF3 supplement groups were dose-dependently increased in that compared to C group (*p* < 0.05) (Figure 2E–G). These finding indicated that PF3 supplement ameliorated skin hydration and the mRNA expression of skin moisturizing-related factors induced by UVB irradiation.

### 3.3. Effects of PF3 on mRNA Expression of Pro-Inflammatory Cytokines (IL-1β, IL-6, and TNF-α); Antioxidant Activities; and Protein Expression of IκBα, NF-κB, and COX-2 in UVB-Irradiated SKH-I Hairless Mice

**Figure 3:**
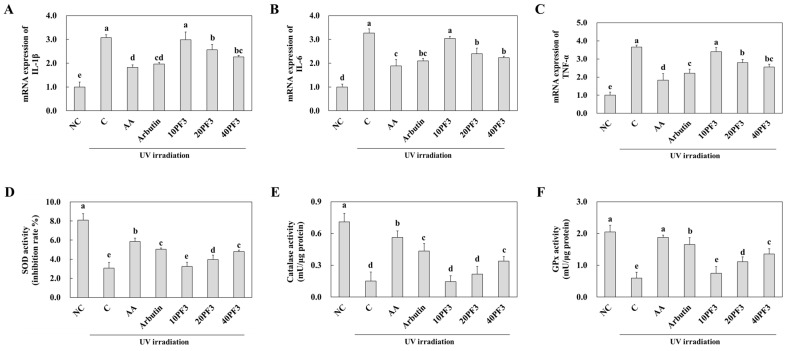
Effect of PF3 on mRNA expression of IL-1β (**A**), IL-6 (**B**), TNF-α (**C**), SOD activity (**D**), catalase activity (**E**), and GPx activity (**F**) in the dorsal skin of UVB-irradiated SKH-I hairless mice. NC, AIN93G without UVB irradiation; C, AIN93G with UVB irradiation; AA, AIN93G supplemented 100 mg/kg body weight (BW) of L-ascorbic acid with UVB irradiation; Arbutin, AIN93G supplemented 100 mg/kg BW of arbutin with UVB irradiation; 10PF3, AIN93G supplemented 10 mg/kg BW of PF3 with UVB irradiation; 20PF3, AIN93G supplemented 20 mg/kg BW of PF3 with UVB irradiation; 40PF3, AIN93G supplemented 40 mg/kg BW of PF3 with UVB irradiation. Data represent the mean ± SD (*n* = 8). Different letters (a–e) represent significant differences at *p* < 0.05, as determined by Duncan’s multiple rang test.

**Figure 4:**
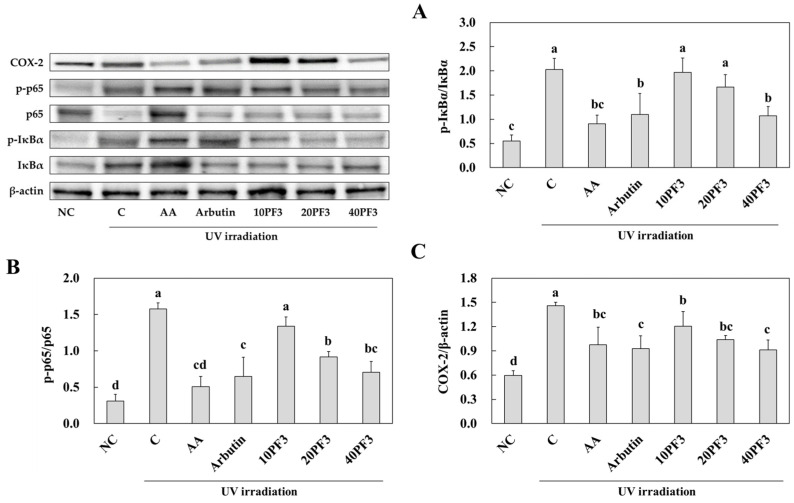
Effect of PF3 on protein expression of p-IκBα/IκBα (**A**), p-p65/p65 (**B**), and COX-2 (**C**) in the dorsal skin of UVB-irradiated SKH-I hairless mice. NC, AIN93G without UVB irradiation; C, AIN93G with UVB irradiation; AA, AIN93G supplemented 100 mg/kg body weight (BW) of L-ascorbic acid with UVB irradiation; Arbutin, AIN93G supplemented 100 mg/kg BW of arbutin with UVB irradiation; 10PF3, AIN93G supplemented 10 mg/kg BW of PF3 with UVB irradiation; 20PF3, AIN93G supplemented 20 mg/kg BW of PF3 with UVB irradiation; 40PF3, AIN93G supplemented 40 mg/kg BW of PF3 with UVB irradiation. Data represent the mean ± SD (*n* = 8). Different letters (a–d) represent significant differences at *p* < 0.05, as determined by Duncan’s multiple rang test.

To investigate the effect of PF3 on mRNA expression of pro-inflammatory cytokines (IL-1β, IL-6, and TNF-α); antioxidant activities; and protein expression of IκBα, NF-κB, and COX-2 of the dorsal skin of UVB-irradiated SHK-I hairless mice. The mRNA expression of IL-1β, IL-6, and TNF-α was significantly increased in the C group compared to the NC group. AA, arbutin, and PF3 supplement groups were significantly decreased in that compared to C group (*p* < 0.05) (Figure 3A–C). However, the antioxidant enzyme activities (SOD, CAT, and GPx) were significantly decreased in the C group compared to the NC group. AA, arbutin, and PF3 supplement groups were significantly increased in those compared to C group (*p* < 0.05) Especially, SOD and GPx activities increased dose-dependently in PF3 (Figure 3D–F). Moreover, protein expression of p-IκBα/ IκBα, p-p65/p65, and COX-2 was significantly increased in the C group compared to the NC group and significantly decreased in AA, arbutin, and PF3 compared to that in C group (*p* < 0.05) (Figure 4A–C). These results indicated PF3 supplement improved inflammation and antioxidant factors induced by UVB irradiation.

### 3.4. Effects of PF3 on mRNA and Protein Expression of Skin Wrinkle Formation-Related Factors in UVB-Irradiated SKH-I Hairless Mice

**Figure 5:**
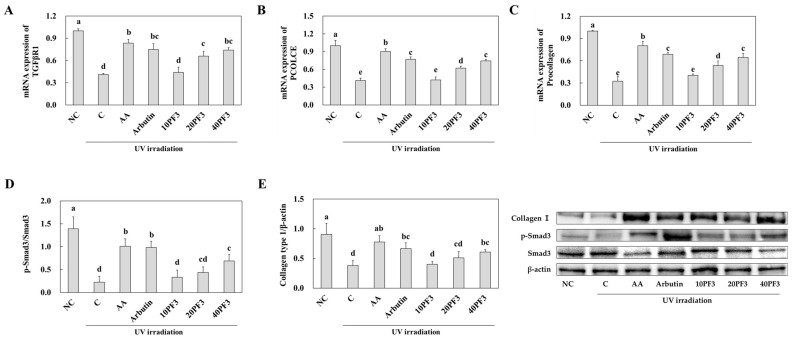
Effect of PF3 on mRNA expression of TGFβR1 (**A**), PCOLCE (**B**), Procollagen (**C**), p-Smad3/Smad3 (**D**), and Collagen type I (**E**) in the dorsal skin of UVB-irradiated SKH-I hairless mice. NC, AIN93G without UVB irradiation; C, AIN93G with UVB irradiation; AA, AIN93G supplemented 100 mg/kg body weight (BW) of L-ascorbic acid with UVB irradiation; Arbutin, AIN93G supplemented 100 mg/kg BW of arbutin with UVB irradiation; 10PF3, AIN93G supplemented 10 mg/kg BW of PF3 with UVB irradiation; 20PF3, AIN93G supplemented 20 mg/kg BW of PF3 with UVB irradiation; 40PF3, AIN93G supplemented 40 mg/kg BW of PF3 with UVB irradiation. Data represent the mean ± SD (*n* = 8). Different letters (a–e) represent significant differences at *p* < 0.05, as determined by Duncan’s multiple rang test.

**Figure 6:**
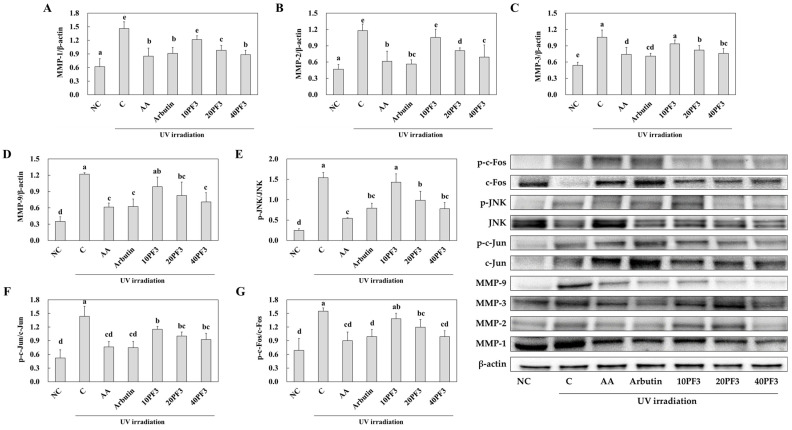
Effect of PF3 on protein expression of MMP-1 (**A**), MMP-2 (**B**), MMP-3 (**C**), MMP-9 (**D**), p-JNK/JNK (**E**), p-c-Jun/c-Jun (**F**), and p-c-Fos/c-Fos (**G**) in the dorsal skin of UVB-irradiated SKH-I hairless mice. NC, AIN93G without UVB irradiation; C, AIN93G with UVB irradiation; AA, AIN93G supplemented 100 mg/kg body weight (BW) of L-ascorbic acid with UVB irradiation; Arbutin, AIN93G supplemented 100 mg/kg BW of arbutin with UVB irradiation; 10PF3, AIN93G supplemented 10 mg/kg BW of PF3 with UVB irradiation; 20PF3, AIN93G supplemented 20 mg/kg BW of PF3 with UVB irradiation; 40PF3, AIN93G supplemented 40 mg/kg BW of PF3 with UVB irradiation. Data represent the mean ± SD (*n* = 8). Different letters (a–d) represent significant differences at *p* < 0.05, as determined by Duncan’s multiple rang test.

To investigate the changes in mRNA and protein expression of skin wrinkle formation-related factors by PF3 supplementation, we analyzed the dorsal skin from UVB-irradiated SHK-I hairless mice. mRNA expression of TGFβR1, PCOLCE, and procollagen was significantly decreased in the C group compared to NC group and was significantly increased in the AA, arbutin, and PF3 supplement groups compared to the C group (*p* < 0.05) (Figure 5A–C). Also, the protein expression of p-Smad3/Smad3 and collagen I was decreased in C group and was significantly increased in the AA, arbutin, and PF3 supplement groups compared to the C group (*p* < 0.05) (Figure 5D,E). However, the protein expression of MMP-1, MMP-2, MMP-3, MMP-9, p-JNK/JNK, p-c-Jun/c-Jun, and p-c-Fos/c-Fos was significantly increased in C group compared to NC group and was decreased in the AA, arbutin, and PF3 supplement groups compared to the C group (*p* < 0.05) (Figure 6A–G). These finding suggested that PF3 supplement improved the skin wrinkle formation-related factors induced by UVB irradiation.

### 3.5. Effects of the PF3 on Nitric Oxide, cAMP Content, and Protein Expression of Skin Melanogenesis-Related Factors in UVB-Irradiated SKH-I Hairless Mice

**Figure 7:**
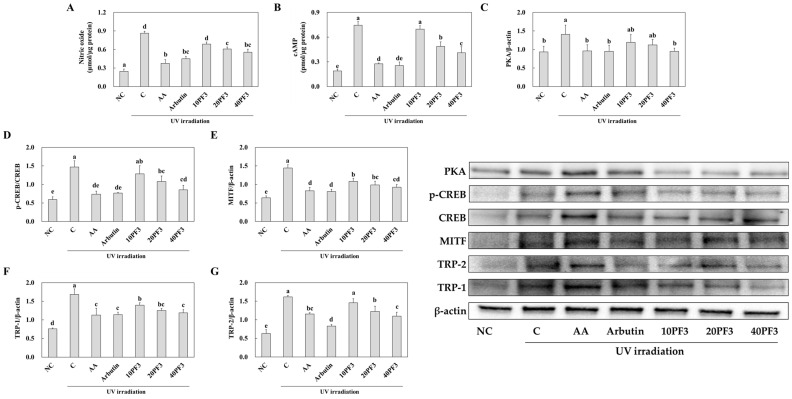
Effect of PF3 on levels of nitric oxide (**A**) and cAMP (**B**), protein expression of PKA (**C**), p-CREB/CREB (**D**), MITF (**E**), TRP-1 (**F**), and TRP-2 (**G**) in the dorsal skin of UVB-irradiated SKH-I hairless mice. NC, AIN93G without UVB irradiation; C, AIN93G with UVB irradiation; AA, AIN93G supplemented 100 mg/kg body weight (BW) of L-ascorbic acid with UVB irradiation; Arbutin, AIN93G supplemented 100 mg/kg BW of arbutin with UVB irradiation; 10PF3, AIN93G supplemented 10 mg/kg BW of PF3 with UVB irradiation; 20PF3, AIN93G supplemented 20 mg/kg BW of PF3 with UVB irradiation; 40PF3, AIN93G supplemented 40 mg/kg BW of PF3 with UVB irradiation. Data represent the mean ± SD (*n* = 8). Different letters (a–e) represent significant differences at *p* < 0.05, as determined by Duncan’s multiple rang test.

To investigate the changes in NO, cAMP content, and protein expression of skin melanogenesis-related factors by PF3 supplementation, we analyzed the dorsal skin from UVB-irradiated SHK-I hairless mice. The NO and cAMP content were significantly increased in the C group compared to the NC group and were significantly decreased in the AA, arbutin, and PF3 supplement groups, except the PF3 10 group, compared to the C group (*p* < 0.05) (Figure 7A). Furthermore, the protein expression of PKA, p-CREB/CREB, MITF, TRP-1, and TRP-2 was significantly increased in C group compared to NC group and was decreased in the AA, arbutin, and PF3 supplement groups compared to the C group (*p* < 0.05) (Figure 6A–G). These finding suggested that PF3 supplement improved the skin melanogenesis-related factors induced by UVB irradiation.

## 4. Discussion

Ultraviolet (UV) radiation exerts a complex array of effects on human skin, ranging from beneficial to potentially harmful. While moderate UV exposure stimulates vitamin D synthesis, essential for bone health and immune function, excessive exposure can lead to both acute and chronic skin damage. Chronically, it contributes to photoaging, manifesting as wrinkles, loss of elasticity, and uneven pigmentation. UV exposure also generates reactive oxygen species, causing oxidative stress and degrading collagen, which further accelerates the aging process [22,23,24]. SKH-1 hairless mice have become a preferred model for studying UVB-induced skin health effects due to their unique characteristics that closely mimic human skin responses. Their skin structure, including epidermal thickness and dermal-epidermal junction, is similar to human skin, allowing for more translatable results, and SKH-1 mice also exhibit comparable photobiological responses to humans, such as erythema, edema, and hyperplasia, following UVB exposure [25,26]. Recent research has focused on the potential of plant-derived ceramides for skin protection and health enhancement. Plant-derived glucosylceramides typically consist of a sphingoid base linked to a fatty acid, with a glucose moiety attached to the primary hydroxyl group of the sphingoid base [3]. The sphingoid base in plant ceramides often varies between species, with common forms including sphingosine, phytosphingosine, and sphinganine [13]. The fatty acid component of plant-derived glucosylceramides can range from C16 to C26 in chain length, with both saturated and unsaturated forms observed [3]. This study, utilizing peach-derived glucosylceramide (PF3), demonstrated its efficacy in increasing the ceramide content in a three-dimensional human skin model and improving skin moisture retention function upon oral administration. These findings suggest that peach-derived glucosylceramide may possess structural or functional similarities to human skin ceramides. While the exact chemical structure of the peach-derived glucosylceramide was not explicitly detailed in the previous study, a review of related literature provides insights into its composition [18,19]. The present study examined whether PF3 supplementation could inhibit the skin damage caused by UVB-irradiated photoaging and oxidative stress through its effects on skin hydration, wrinkles, and melanogenesis.

UV radiation can alter the expression of the genes involved in skin hydration, such as those regulating hyaluronic acid synthesis and ceramide production. As a consequence, UV-exposed skin often exhibits signs of dryness, flakiness, and reduced elasticity, contributing to premature aging and compromised skin health. Skin hydration, which plays a crucial role in maintaining epidermal barrier function and overall skin health, is a complex process involving various genes and molecular pathways that contribute to skin structure, moisture retention, and barrier integrity. Hyaluronic acid synthases (HAS1-3) are key enzymes responsible for the production of hyaluronic acid, a major component of the extracellular matrix that contributes significantly to skin hydration. These genes produce hyaluronic acid of varying molecular weights, which play crucial roles in moisture retention and skin viscoelasticity, and the LCB1 (long-chain base 1) gene is involved in the biosynthesis of ceramides, which are essential components of the skin barrier. Ceramides play a vital role in preventing transepidermal water loss and maintaining skin hydration. Proper functioning of the LCB1 gene is necessary for adequate ceramide production and, consequently, skin barrier function. DEGS1 is another gene important for skin barrier function and hydration. This gene is involved in the final step of ceramide biosynthesis, catalyzing the formation of ceramides with specific fatty acid compositions [27,28]. In addition, NF-κB activation can modulate the expression of genes involved in skin barrier maintenance and repair. Excessive MMP activity can lead to the degradation of structural proteins like collagen and elastin, potentially compromising skin hydration and barrier function. COX-2 is an enzyme involved in the production of prostaglandins, which play a role in inflammation and skin barrier function. The regulation of COX-2 expression can impact skin hydration and barrier integrity, particularly in the context of inflammatory skin conditions [29,30,31]. In this study, PF3 increased skin hydration and mRNA expression of HAS1-3, LCB1 (SPT), and DEGS1 and decreased the protein expression of p-IκB/IκB, MMPs, and COX-2. These results recommend that PF3 could manage skin hydration via the upregulation of HA and ceramide synthesis and downregulation of the NF-κB and MMP pathways.

The formation of skin wrinkles is a complex process involving multiple molecular pathways and factors. Recent research has shed light on the roles of specific signaling molecules and enzymes in this process, particularly the involvement of the JNK (c-Jun N-terminal kinase) pathway, transcription factors c-Fos and c-Jun, and matrix metalloproteinases (MMPs). The JNK signaling cascade plays a crucial role in cellular responses to various stressors, including UV radiation, which is a major contributor to skin aging and wrinkle formation. When activated, JNK becomes phosphorylated (p-JNK), leading to the subsequent activation of downstream targets. Transcription factors c-Fos and c-Jun, components of the AP-1 complex, are key regulators of gene expression in response to various stimuli, including those that promote skin aging. The phosphorylation of these factors (p-c-Fos and p-c-Jun) enhances their activity. Matrix metalloproteinases (MMPs) are enzymes that degrade extracellular matrix components, including collagen and elastin, which are essential for maintaining skin structure and elasticity. Increased expression and activity of MMPs, particularly MMP-1, MMP-3, and MMP-9, have been linked to the breakdown of dermal collagen and elastin fibers, contributing to the formation of wrinkles. The interplay between these factors creates a cascade of events leading to skin aging and wrinkle formation. UV radiation and other stressors can activate the JNK pathway, leading to increased phosphorylation of c-Fos and c-Jun. This, in turn, promotes the transcription of MMP genes, resulting in elevated levels of these enzymes in the skin. The subsequent degradation of collagen and elastin by MMPs weakens the skin’s structural integrity, ultimately manifesting as visible wrinkles [32,33,34]. In this study, PF3 decreased the protein expression of p-JNK/JNK, p-c-Fos/c-Fos, p-c-Jun/c-Jun, and MMPs, while the PF3 complex increased the protein expression of p-Smad3/Smad3. These results suggest that PF3 could protect skin wrinkle formation via the upregulation of Smad3 and downregulation of the JNK, c-Fos, c-Jun, and MMPs pathways.

When skin is exposed to UV radiation, it triggers the activation of the cAMP-dependent protein kinase A (PKA) pathway. This activation results in the phosphorylation of the cAMP response element-binding protein (CREB), a crucial transcription factor in the melanogenesis process. Phosphorylated CREB then translocates to the nucleus, where it binds to the promoter region of the microphthalmia-associated transcription factor (MITF) gene, enhancing its expression. MITF plays a central role in melanogenesis by acting as a master regulator of melanocyte development and function. Once upregulated, MITF binds to the promoter regions of key melanogenic enzymes, including tyrosinase, tyrosinase-related protein-1 (TRP-1), and tyrosinase-related protein-2 (TRP-2). This binding promotes the transcription of these enzymes, which are essential for melanin synthesis. Tyrosinase catalyzes the initial and rate-limiting steps of melanin production, while TRP-1 and TRP-2 are involved in the later stages of melanogenesis. TRP-1 stabilizes tyrosinase and increases its catalytic activity, while TRP-2, also known as dopachrome tautomerase, isomerizes dopachrome to 5,6-dihydroxyindole-2-carboxylic acid (DHICA) in the eumelanin synthesis pathway [35,36,37]. Arbutin is frequently used in skin whitening experiments for several key reasons. It effectively inhibits melanin production by suppressing tyrosinase activity, a primary mechanism in skin whitening. Compared to hydroquinone, arbutin is known for its better safety profile and lower skin irritation, making it suitable for long-term use in whitening products. Its antioxidant properties contribute to reducing oxidative stress in the melanin production pathway, offering additional skin protection benefits [38,39]. In this study, PF3 decreased the NO and cAMP contents and protein expression of p-PKA/PKA, p-CREB/CREB, MITF, TRP-1, and TRP-2. These results indicated that PF3 could protect skin melanogenesis via suppressing of the PKA/CREB/MITF/TRP-1/TRP-2 pathway.

## 5. Conclusions

The research outcomes indicate that PF3 supplementation shows considerable potential in mitigating skin photoaging. PF3 influences crucial molecular mechanisms related to skin moisture retention, wrinkle development, and pigment production. By addressing these key factors, PF3 emerges as a promising agent for preserving skin health and counteracting the detrimental impacts of UVB exposure. The study’s results suggest that incorporating PF3 into skincare regimens could offer a proactive approach to combating the signs of skin aging caused by environmental stressors, particularly UV radiation. While further investigation is recommended to confirm these results in clinical trials, these initial findings highlight PF3’s potential as a valuable tool in preventive dermatological care.

## Data Availability

The original contributions presented in this study are included in the article/Supplementary Materials. Further inquiries can be directed to the corresponding author.

## Supplementary Materials

The following supporting information can be downloaded at: https://www.mdpi.com/article/10.3390/foods13233824/s1, Figure S1. Effect of PF3 on mRNA expression of HAS1 (A), HAS2 (B), HAS3 (C), LCB1 (D), DEGS1 (E), and Elastin (F), IL-1β production (G), IL-6 production (H), TNF-α production (I), SOD activities (J), Catalase activity (K), GPx activity (L), and hyaluronic acid (M) in UVB-irradiated HaCaT cells. NC, without UVB irradiation; C, UVB irradiation; AA, 100 μg/mL L-ascorbic acid with UVB irradiation; 50, 50 μg/mL PF3 with UVB irradiation; 150, 150 μg/mL PF3 with UVB irradiation; 300, 300 μg/mL PF3 with UVB irradiation. Data represent the mean±SD. Different letters (a–e) represent significant differences at *p* < 0.05, as determined by Duncan’s multiple rang test. Figure S2. Effect of PF3 on protein band (A), protein expression of LASS4 (B), p-IκBα/IκBα (C), p-p65/p65 (D), and COX-2 (E) in UVB-irradiated HaCaT cells. NC, without UVB irradiation; C, UVB irradiation; AA, 100 μg/mL L-ascorbic acid with UVB irradiation; 50, 50 μg/mL PF3 with UVB irradiation; 150, 150 μg/mL PF3 with UVB irradiation; 300, 300 μg/mL PF3 with UVB irradiation. Data represent the mean ± SD. Different letters (a–e) represent significant differences at *p* < 0.05, as determined by Duncan’s multiple rang test. Figure S3. Effect of PF3 on mRNA expression of TGFβR1 (A), PCOLCE (B), and procollagen (C) in UVB-irradiated Hs27 cells. NC, without UVB irradiation; C, UVB irradiation; AA, 100 μg/mL L-ascorbic acid with UVB irradiation; 50, 50 μg/mL PF3 with UVB irradiation; 150, 150 μg/mL PF3 with UVB irradiation; 300, 300 μg/mL PF3 with UVB irradiation. Data represent the mean±SD. Different letters (a–e) represent significant differences at *p* < 0.05, as determined by Duncan’s multiple rang test. Figure S4. Effect of PF3 on protein band (A), protein expression of MMP-1 (B), MMP-2 (C), MMP-3 (D), MMP-9 (E), p-JNK/JNK (F), p-c-Fos/c-Fos (G), p-c-Jun/c-Jun (H), and p-Smad3/Smad3 (I) in UVB-irradiated Hs27 cells. NC, without UVB irradiation; C, UVB irradiation; AA, 100 μg/mL L-ascorbic acid with UVB irradiation; 50, 50 μg/mL PF3 with UVB irradiation; 150, 150 μg/mL PF3 with UVB irradiation; 300, 300 μg/mL PF3 with UVB irradiation. Data represent the mean±SD. Different letters (a–e) represent significant differences at *p* < 0.05, as determined by Duncan’s multiple rang test.
