# Supplementary material for: Effect of Ceramides Derivatives from the Peach on Skin Function Improvement in UV-Irradiated Hairless Mice
Source: Foods. 2024 Nov 27;13(23):3824. doi: 10.3390/foods13233824 (PMC11640303; doi:10.3390/foods13233824)
Supplement: Supplementary file 1 [file foods-13-03824-s001.zip › foods-3279912-supplementary.pdf]

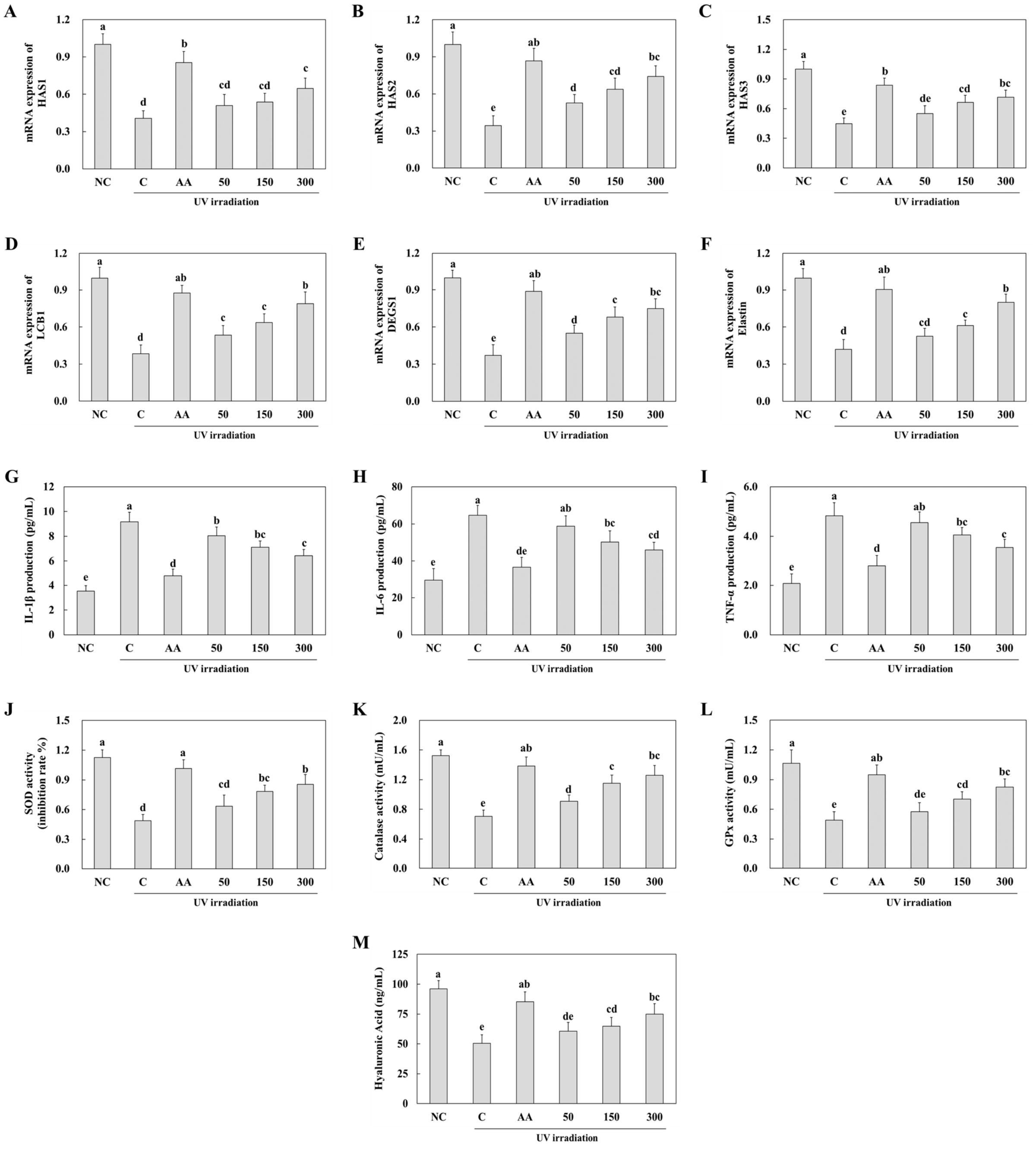

Figure S1. Effect of PF3 on mRNA expression of HAS1 (A), HAS2 (B), HAS3 (C), LCB1 (D), DEGS1 (E), and Elastin (F), IL-1β production (G), IL-6 production (H), TNF-α production (I), SOD activities (J), Catalase activity (K), GPx activity (L), and hyaluronic acid (M) in UVB-irradiated HaCaT cells. NC, without UVB irradiation; C, UVB irradiation; AA, 100 μg/mL L-ascorbic acid with UVB irradiation; 50, 50 μg/mL PF3 with UVB irradiation; 150, 150 μg/mL PF3 with UVB irradiation; 300, 300 μg/mL PF3 with UVB irradiation. Data represent the mean±SD. Different letters (a-e) represent significant differences at $p<0.05$, as determined by Duncan's multiple rang test.

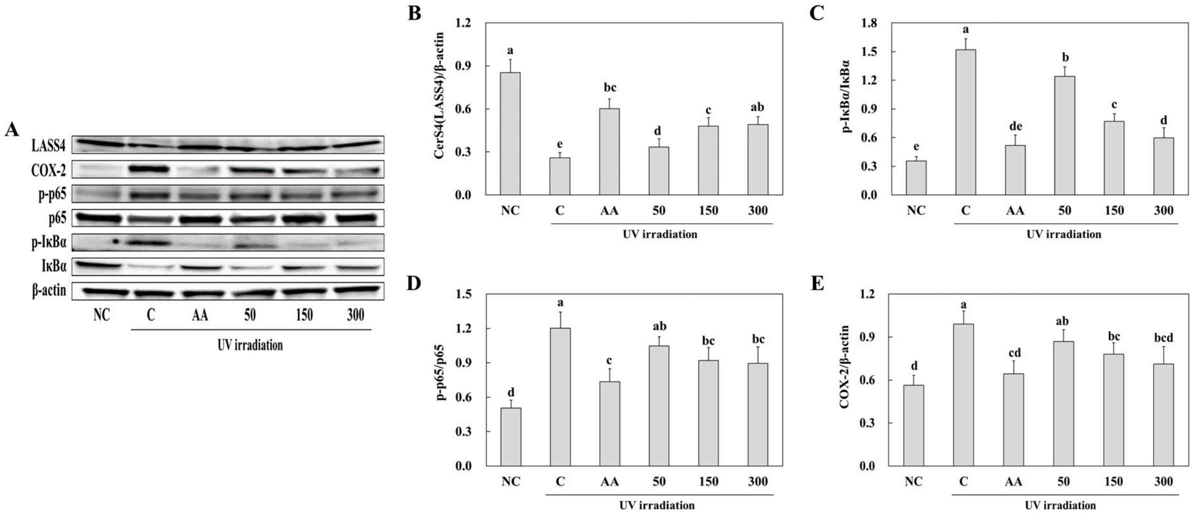

Figure S2. Effect of PF3 on protein band (A), protein expression of LASS4 (B), p-IκBα/IκBα (C), p-p65/p65 (D), and COX-2 (E) in UVB-irradiated HaCaT cells. NC, without UVB irradiation; C, UVB irradiation; AA, 100 μg/mL L-ascorbic acid with UVB irradiation; 50, 50 μg/mL PF3 with UVB irradiation; 150, 150 μg/mL PF3 with UVB irradiation; 300, 300 μg/mL PF3 with UVB irradiation. Data represent the mean±SD. Different letters (a-e) represent significant differences at $p<0.05$, as determined by Duncan's multiple rang test.

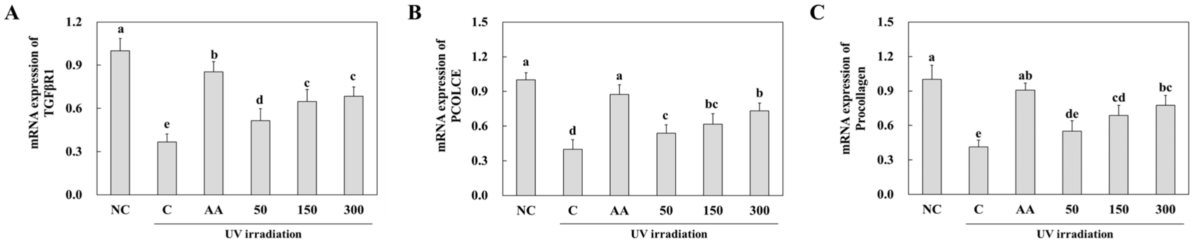

Figure S3. Effect of PF3 on mRNA expression of TGFβR1 (A), PCOLCE (B), and procollagen (C) in UVB-irradiated Hs27 cells. NC, without UVB irradiation; C, UVB irradiation; AA, 100 μg/mL L-ascorbic acid with UVB irradiation; 50, 50 μg/mL PF3 with UVB irradiation; 150, 150 μg/mL PF3 with UVB irradiation; 300, 300 μg/mL PF3 with UVB irradiation. Data represent the mean±SD. Different letters (a-e) represent significant differences at $p<0.05$, as determined by Duncan's multiple rang test.

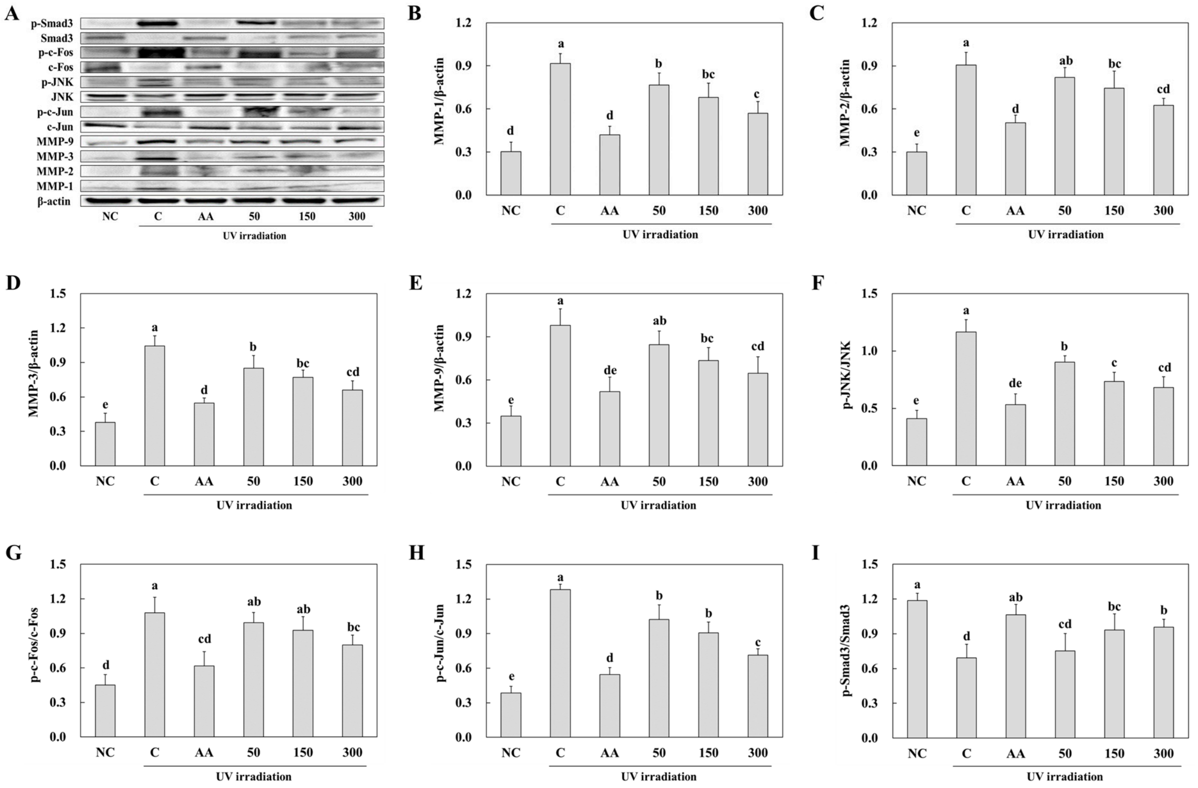

Figure S4. Effect of PF3 on protein band (A), protein expression of MMP-1 (B), MMP-2 (C), MMP-3 (D), MMP-9 (E), p-JNK/JNK (F), p-c-Fos/c-Fos (G), p-c-Jun/c-Jun (H), and p-Smad3/Smad3 (I) in UVB-irradiated Hs27 cells. NC, without UVB irradiation; C, UVB irradiation; AA, 100 µg/mL L-ascorbic acid with UVB irradiation; 50, 50 µg/mL PF3 with UVB irradiation; 150, 150 µg/mL PF3 with UVB irradiation; 300, 300 µg/mL PF3 with UVB irradiation. Data represent the mean±SD. Different letters (a-e) represent significant differences at $p<0.05$, as determined by Duncan's multiple rang test.
